# Frequency and volumetry of infraorbital ethmoid cells (Haller cells) on cone-beam computed tomograms (CBCT) of the mid-face

**DOI:** 10.3205/iprs000109

**Published:** 2017-04-11

**Authors:** Reinhard E. Friedrich, Meike Fraederich, Gerhard Schoen

**Affiliations:** 1 Department of Oral and Cranio-Maxillofacial Surgery, University Medical Center Hamburg-Eppendorf, Hamburg, Germany; 2 Department of Medical Biometry and Epidemiology, University Medical Center Hamburg-Eppendorf, Hamburg, Germany

**Keywords:** Haller cell, cone-beam computed tomography (CBCT), radiological anatomy, volumetric measurement, paranasal sinuses

## Abstract

**Objective:** The aim of this study was to determine the frequency and morphology of Haller cells using a new radiological technique that allows examination of the facial skull.

**Material and methods:** In a single center retrospective cohort study the cone-beam computed tomography (CBCT) volume data of the maxillary sinus of 199 patients were analysed (398 paranasal sinuses). If Haller cells were found, their capacity was determined. If orthopantomograms (OPG) were taken in a narrow time frame around the CBCT investigation, the rate of correspondence of the radiological findings was determined. The correlation between frequency of Haller cells and age and gender was calculated.

**Results:** Out of 199 patients 47 showed at least one Haller cell (23.62%). The total number of Haller cells was 64. Correspondence was rare in the same case between the results from different examination procedures with respect to the target object.

**Conclusions:** Bony variants of paranasal sinuses can be analysed using CBCT as an alternative to computed tomography (CT), whereas OPG images do not reliably detect Haller cells.

## Zusammenfassung

**Einleitung:** Ziel der Untersuchung war es, die Häufigkeit und Morphologie von Haller-Zellen mit Hilfe einer neuen radiologischen Technik zu bestimmen, die für die skelettale Darstellung des Kieferbereichs entwickelt worden ist.

**Material und Methode:** In einer retrospektiven Kohortenstudie wurden die digitalen Volumentomogramme (DVT) der Kieferhöhlen von 199 Patienten ausgewertet (398 paranasale Sinus). Beim Nachweis von Haller-Zellen wurde deren Volumen bestimmt. Falls in zeitlicher Nähe zum Anfertigungsdatum der DVT ein Orthopantomogramm (OPG) des jeweiligen Patienten angefertigt worden war, wurde die Übereinstimmungsrate der Befunde des Nachweises oder Ausschlusses von Haller-Zellen für diese Patientengruppe bestimmt. Die Korrelation der Häufigkeit des Auftretens von Haller-Zellen in Abhängigkeit von Alter und Geschlecht wurde berücksichtigt.

**Ergebnisse:** Mindestens eine Haller-Zelle wurde bei 47 von 199 Patienten diagnostiziert (23,62%). Die Gesamtzahl der Haller-Zellen war 64. Die Übereinstimmung des radiologischen Befundes hinsichtlich der Zielstruktur dieser Analyse war zwischen den beiden radiologischen Untersuchungsverfahren sehr gering. Nach unserer Analyse ist das OPG für die Beurteilung von Haller-Zellen ungeeignet.

**Fazit:** Knöcherne Variationen der Nasennebenhöhlen lassen sich mit der DVT sehr gut darstellen. Diese Untersuchungstechnik stellt für diese Fragestellung eine Alternative zur Computertomographie dar. Mit dem OPG können keine verlässlichen Aussagen zum Nachweis von Haller-Zellen getroffen werden.

## Introduction

The paranasal sinuses are of significant practical interest as problems in this area are common. They are composed of a complex three-dimensional configuration in a subsurface site with a considerable range of variation [1], [2]. The ethmoidal complex is the central part of the paranasal sinuses and comprises, on average, 8–15 air cells. These are clearly separated into anterior or posterior ethmoid cells by the course of the lamina of the middle concha [3], [4]. Ethmoid cells that advance into the orbital floor are colloquially called “Haller Cells”. The correct academic term according to the “International Conference on Sinus Disease” is “infraorbital ethmoid cell (Haller’s cell)” or *“cellulae ethmoidales infraorbitales”* [3]. They are named after Albrecht von Haller (1708–1777), a Swiss polymath who studied and worked in Germany, the Netherlands, France, and Switzerland [5], [6] and conducted anatomical studies on the skull. In 1743 he was the first to describe “orbital air cells” in macerated skulls. He commented on them several times again during the following years [5], [6]. Finally, after decades of inobservance the eponym “Haller cell” was naturalized in the medical literature when interest in their study re-emerged, nowadays complemented by the use of new technologies like endoscopy and diagnostic radiology [5], [6].

### Development

To date it is not known whether Haller cells develop during the embryonic period or later in life [7], [8]. Chimpanzees and possibly orang-utans show pneumatization in the ethmoid bone similar to humans, perhaps due to their upright posture. The ethmoid cells are unique among the different sinuses with their paper-thin bony walls, allowing them to migrate more easily in adjacent extramural bone structures, which could explain their miscellaneous bony variants [9]. In one study, Haller cells were found more frequently in patients with sinunasal osteoma than in controls. So it was assumed that the same environmental factors may affect formation of osteoma and Haller cells [10]. On the other hand, huge pneumatization of the paranasal sinuses was occasionally reported as a variant without associated pathology. In this case, the unilateral Haller cell was also large [11]. 

### Clinical relevance

Haller cells differ in size and number and may trigger different afflictions. The focus of interest is to identify these cells with appropriate instruments, preferably in a simple and non-invasive way, with minimum radiation, low cost, and the ability to distinguish between several differential diagnoses. 

Haller cells are often an incidental finding and not a disease pattern per se [12], [13]. However, just the identification of the anatomical alteration can be very important for planning surgical interventions to avoid complications or a second operation [14], [15]. Their pathophysiological importance results from their narrowing of the pathway of the maxillary ostium or the infundibulum ethmoidale [3], [12], [16]. Their impact depends on their size and location [13], [16], [17].

Particularly large cells have been found to cause sinusitis and sinusal headache [12], [13], [17]. On the other hand, even small Haller cells can generate these complaints. Therefore, there is no general interaction between the size of cells and symptoms [12], [16], [17]. In diverse documented cases Haller cells caused many different diseases like headache or facial pain [12], [18], [19], or a mucocele inside the cell was found [20], with inflammation [21] or associated orbital edema [22]. In such cases surgical interventions may be expedient [13], [20], [22]. 

Recent studies have focused on the inter-relationship between anatomical variants of the paranasal sinuses and allergic disorders. There was no statistical correlation with allergic rhinitis [23], but there was a correlation with the presence or development of chronic rhinosinusitis [24], [25], [26].

Earwaker summed up the relevance of anatomical variations including Haller cells:

- they may block drainage routes,
- they may obstruct distal regions, impairing the passage of endoscopes,
- they can be a focus for occult diseases,
- they can increase the risk of failure in endoscopic interventions [27].

### Imaging of maxillary sinus and adjacent osseous structures

The first body-imaging examination in a dental or maxillofacial-surgical praxis is usually performed with an orthopantomogram (OPG), which produces a distorted two-dimensional image. When further information is needed, computed tomography (CT) is often arranged, with the associated time delay, higher radiation dose and potential loss of information due to artifacts. Magnetic resonance imaging (MRI), endoscopy, or ultrasonic examination may be helpful for specific indications. In the majority of cases conventional anterior-posterior radiographs of the midfacial region are unnecessary today due to the superiority of representation of the region of interest by the use of radiological sectional imaging techniques.

Meanwhile cone beam computed tomography (CBCT) has been introduced in diagnostic imaging in the fields of dentistry, maxillofacial surgery, and otorhinolaryngology as a digital, three-dimensional radiographic technique that provides isometric images in all three planes. CBCT involves less radiation exposure, is easily performed in an outpatient office, and is economical due to its lower costs compared to conventional CT examination [28], [29]. CBCT is now recommended for further examination of the maxillary sinus when two-dimensional radiographs do not provide clarification, or for follow-up examination [29], [30], [31]. The incidence of Haller cells has been repeatedly investigated on the basis of CT data, but to our knowledge only rarely on CBCT images [32], [33], [34], [35] and also in three studies using OPG radiographs [2], [27], [34] (for review of recent CT studies see [34]). We therefore investigated the frequency and morphology of Haller cells in CBCT images, added a volumetric measurement, differentiated cells from simple crests, and compared our results to OPG images if available. Finally, we compared the results obtained using cross-sectional imaging techniques (CT, CBCT).

## Material and methods

We here present a retrospective single-center cohort study. In this retrospective study we analysed CBCT data from 398 paranasal sinus of 199 patients investigated from January 2013 to March 2014. Patients underwent X-ray examination for different indications in the outpatient office of the Department of Oral and Cranio-Maxillofacial Surgery at the University Hospital of Hamburg-Eppendorf.

### Patients

Patients with known or suspected surgical intervention in the region of the mid-face were excluded, as well as patients with known or suspected traumatic or neoplastic alterations. Radiographs with questionable quality or with artifacts were also excluded from investigation. The medical charts of all patients were studied. Their age at time of examination and gender were recorded.

### Haller cells on radiographs

CBCT images were then analysed for the presence of infraorbital ethmoid cells. However, there is as yet no general definition of the so-called Haller cell [16]. In our study we followed the definition of Haller cells specified by Simeunovic [6]: ethmoid cells that advance in the orbital floor or the roof of the maxillary sinus, respectively, as far as the vicinity of the maxillary ostium, which may build the lateral wall of the infundibulum. We did not include cells located in the infundibulum or formations originating from posterior ethmoid cells.

**Figure 1:**
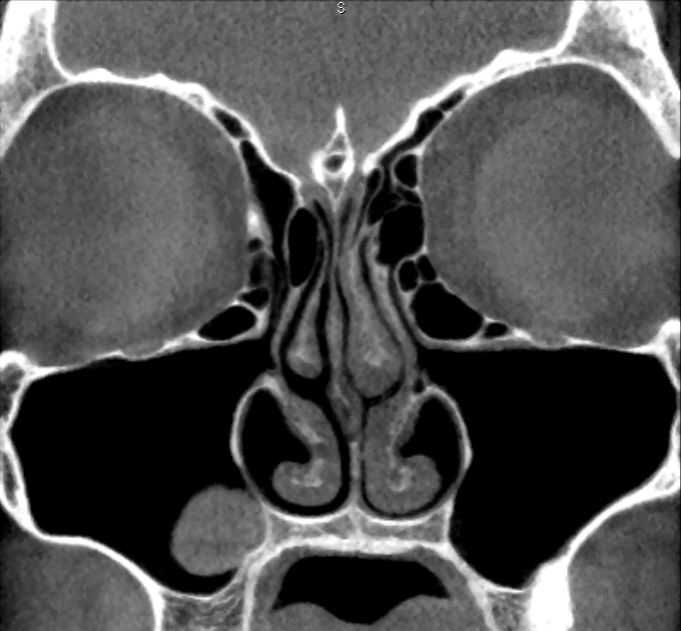
Bilateral Haller cells on coronal plane of CBCT

**Figure 2:**
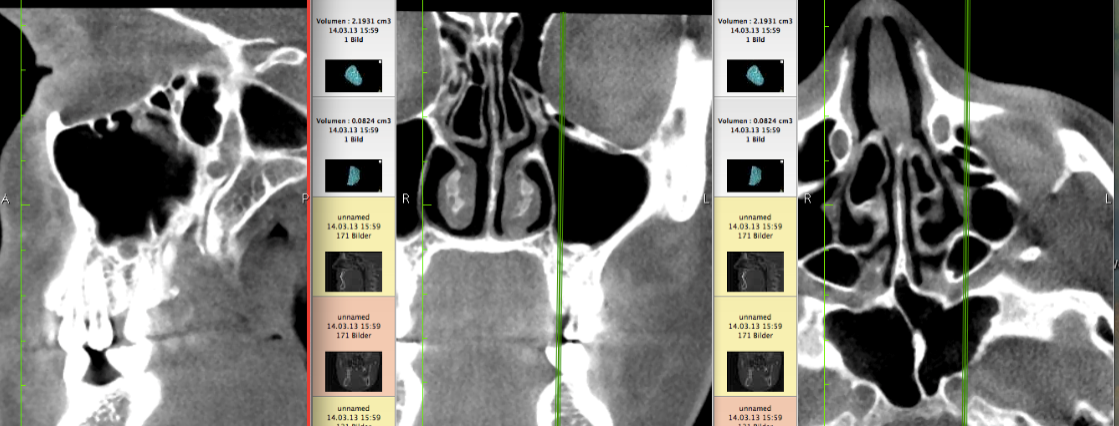
Simple bone crests mimicking Haller cell in coronal plane on the left side. The crest is clearly identified on further planes perpendicular to the coronal aspect.

**Figure 3:**
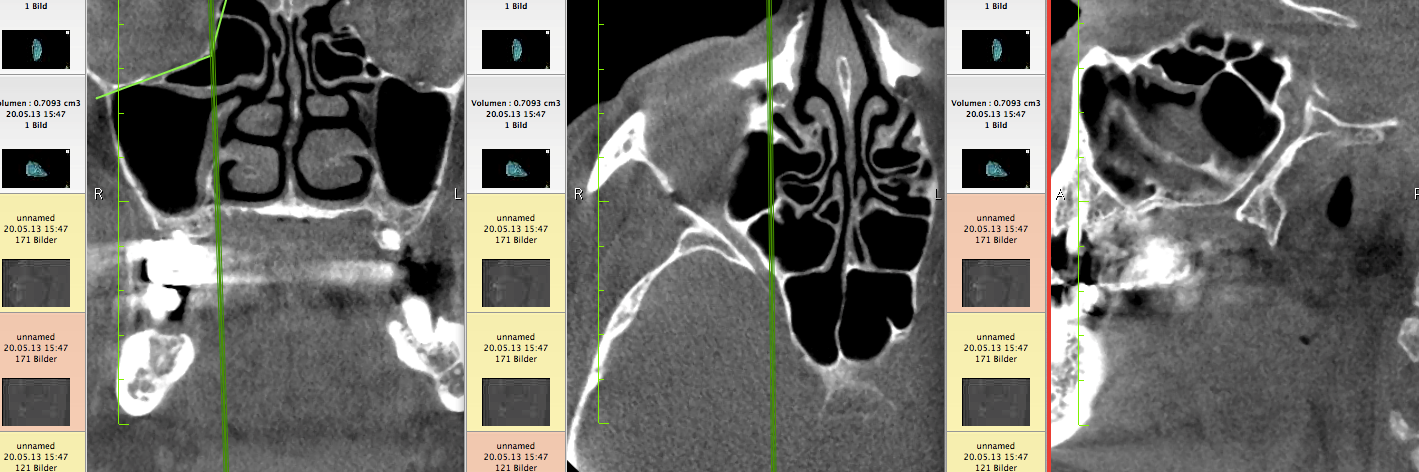
Haller cell in coronal plane and verified in axial and sagittal planes

First, the coronal planes were assessed, as this is a common approach in most published studies [36] (Figure 1 (Fig. 1)). In a second step we inspected the axial and sagittal plane to differentiate Haller cells from simple bone crests. Bone crests often mimic complete cells when only viewed in one plane or in summarized two-dimensional radiographs (Figure 2 (Fig. 2), Figure 3 (Fig. 3)). 

**Figure 4:**
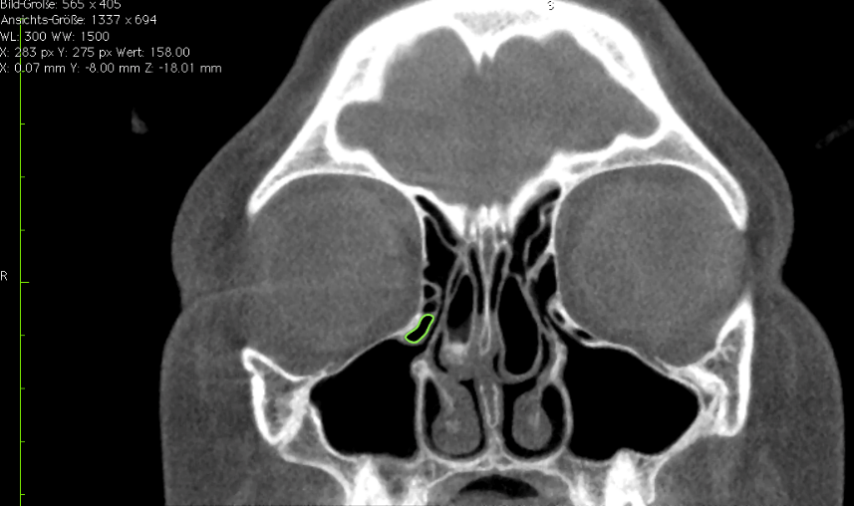
Measurement of Haller cell in coronal plane

**Figure 5:**
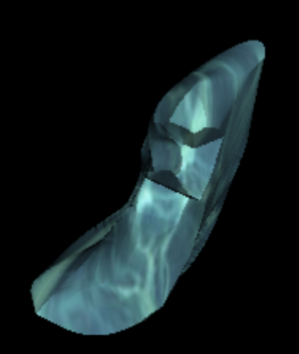
Three-dimensional reconstruction of total volume measurement of Haller cell

Third, we carried out a volumetric measurement (Figure 4 (Fig. 4), Figure 5 (Fig. 5)). If Haller cells were found, we measured the whole cell (total volume) and in addition only the portion located directly beneath the orbital floor (partial volume). In order to define partial volume on radiographs, a perpendicular line was drawn on the medial orbital wall crossing the most medial and cranial point of the orbit floor. The partial volume of infraorbital ethmoidal cells was defined as the volume lateral to this line and adjacent to the orbital floor.

If patients underwent an OPG within one year before or after CBCT examination, we also analysed these images to determine whether Haller cells can be detected reliably, as OPG is a common and often carried out examination in dental and maxillofacial surgical praxis. We compared the results obtained with OPG and our CBCT analyses, but only in a descriptive way. We therefore followed the assessment criteria for Haller cells reported by Ahmad et al. that were used in OPG studies [37], [38], [39]. 

### Calculation aids

All data were then collated in an Excel™ table. Statistical analysis was performed using R Vision 3.1.1™ [40], R-package 1me4 version 1.1-8™ [41] and R-package effects version 3.0-2™ [42]. Differences were considered to be significant at a level of p<0.05.

### Technical data

We used the stationary CBCT scanner 3D Accuitomo 170™ (Morita MFG. Corp., Kyoto, Japan) for CBCT imaging. OPGs were taken using the same OPG scanner, i.e. Veraview IC5 HD™ (Morita). We used i-Dixel 2.0™ (Morita) software for our preselection of CBCT images and for analysis of all matching OPG images. CBCT DICOM data were then analysed using Osirix™ freeware, which allows evaluation of CBCT in detail in all three planes and three-dimensional measurement of structures. 

## Results

We studied the CBCT images of 199 patients. A total of 398 orbito-ethmoidal regions were evaluated. Regions of interest were completely visible in all images. One hundred and seven patients were female, 92 were male. Age ranged from 7 to 96 years with a mean age of 41.58 years. Out of 199 patients 47 (23.62%) showed at least one and a maximum of three Haller cells. The total number of Haller cells was 64. Patients with Haller cells ranged in age from 8 to 96 years; mean age was 40.13 years. Twenty-one patients with Haller cells were female (minimum 12 years to maximum 96 years, mean age was 42.14 years), and 26 were male (minimum 8 years to maximum 71 years, mean age was 38.5 years).

All Haller cells were clearly limited by narrow, continuous radiopacities equivalent to bone. The internal structure of the cells was in all cases completely isodense to air. All Haller cells were measured volumetrically in total. The portion seated beneath the orbital floor (i.e. the component that presents the defining part of these cells) was also measured separately. 

Individual total/partial defining volumes ranged from a minimum 0.002 cm^3^/0.0015 cm^3^ to a maximum 2.547 cm^3^/1.107 cm^3^ with a mean value of 0.347 cm^3^/0.095 cm^3^ in females, and in males from a minimum 0.0029 cm^3^/0.0029cm^3^ to a maximum 1.473 cm^3^/0.805 cm^3^ with a mean value of 0.175 cm^3^/0.077 cm^3^, respectively.

There was no significant statistical correlation between age nor gender and presence or morphology of Haller cells in our evaluation.

We also investigated the appearance of Haller cells on OPG if available and compared the results to the three-dimensional imaging. Thirty cases had undergone CBCT and OPG examination.

Following the criteria of Ahmad et al. [37], we found: 

A total of 13 matches between OPG and CBCT images with, in detail:

- No Haller cell in OPG and no Haller cell in CBCT in eight cases,
- Haller cell in OPG and corresponding cell in CBCT in five cases.

On the other hand there were 17 cases where findings did not match, with, in detail:

- No Haller cell in OPG but in CBCT in two cases,
- Haller cell in OPG but not in CBCT in 15 cases.

We therefore cannot support the opinions of several authors who stated that OPG is an adequate imaging technique for detecting Haller cells [37], [38], [39]. 

## Discussion

Our results concerning the frequency of Haller cells are consistent with results of former CT studies [18], [43]. We found no statistical correlation of frequency or morphology with age or gender. To our knowledge this is the first study to compare the appearance of Haller cells in OPG and CBCT images. We cannot recommend using OPG images for the detection of Haller cells, as there was only a small degree of congruence between findings in OPG compared to CBCT images. Furthermore, this is the first study to differentiate partial and total volume of Haller cells. There are considerable variations in volume of Haller cells both concerning partial and total volume. The partial volume of Haller cells as defined in this study ranged from constituting only a small part of a cell with larger extension to the medial orbital region, but could also constitute the whole volume, if the cell showed no extension beyond the defining vertical line crossing the medial border of the inferior orbit.

In our literature search we found remarkably variable data and information about the frequency of Haller cells on CT, ranging from 2.7% to 60% [35], [44] and even 68% [34]. The frequency in our study was roughly in the middle of the spectrum of published values.

However, there are major differences between previous study designs. Haller cells were investigated in cadaver studies [1], or in CT [15], [16], [25], [27], [45], [46], OPG [37], [38], [39], or CBCT studies [32], [33], [34], [35]. In addition, technological progress means that radiological devices deliver much more detailed images today compared to publications performed earlier or with greater layer thickness of the respective X-ray technology [15]. In addition, some studies only included coronal planes in their analysis, as often recommended [36]. In our experience it is not always possible to discriminate cells from simple crests accurately by only inspecting one plane. For example, Khojastepour et al. explicitly defined orbitoethmoidal cells only on coronal views of CBCT images. It is worth highlighting the extraordinarily high percentage of Haller cells of their study group with respect to their deliberate limitation of the CBCT’s evaluation possibilities [34]. On the other hand, Capelli and Gatti detected Haller cells in 45.7% of their study of mucosal thickening and association of radiological findings in chronic rhinosinusitis patients. These authors declared anatomic variants such as Haller cells to be not significantly associated with symptoms of chronic rhinosinusitis. No definition of Haller cells is given in their study and their published figure of a Haller cell is shown in the coronal plane only [33].

Indeed, there has been no consistent definition of Haller cells in the literature since their discovery [36]. The verification and comparability of data are made more difficult in the case of poor definition of an item. Several authors did not even document the definition adopted in their study [10], [25]. It is clear that different definitions create different study results. This was actually shown by Perez-Pinas et al. [44]. Different surveys studied miscellaneous patient groups varying in age, diseases, or for example ethnic groups [43], [46]. Some authors concluded that anatomical variations of the paranasal sinuses are progressively acquired with maturity [7]. We were not able to confirm or refute this conclusion due to the small sample size in our study, especially in terms of younger patients.

Another limitation of the current study is that it involved a preselected study group. Only patients presenting to the outpatient office were included, and observation bias might be possible. We could not compare the results to a control group due to the use of ionizing radiation as a prerequisite of the study. As in former studies, our study did not include a high number of cases. This may affect the statistical evaluation. 

We did not record the individual indication for CBCT beyond the exclusion criteria. Previous studies showed no statistical correlation between the presence of Haller cells and disorders in general. As all of the Haller cells we found seemed to be asymptomatic, there may be anatomical differences compared with symptomatic cells that we could not detect using an imaging technique that is restricted to visualizing hard tissues. Associations between the thickness of the sinus mucosa and the presence of Haller cells were noted [34], however, the clinical significance of this correlation is uncertain [47], [48].

The only other detailed survey concerning the detection of Haller cells on CBCT radiographs by Mathews et al. was performed with only 50 patients and was based on their own definition of Haller cells that had not been implemented previously [35]. Therefore, we cannot compare our results with these because the definition criteria of the radiological target do not match between the two groups. The frequency of Haller cells was 60% in their study group. The authors attribute the high number of Haller cells to the potentially higher slice intervals of CT used in other studies that could contribute to missing small cells. Only coronal views are presented to illustrate the radiological findings. Volume was not determined, but only the size (qualitatively divided into small, medium, and large).

We recommend conducting further studies with CBCT images to elucidate our results more clearly, especially concerning variations of the paranasal sinuses in children. CBCT allows detailed analysis and is an elegant and non-invasive method for visualizing the ethmoid air cells.

## Conclusions

The ethmoid sinus is a complex bony structure known to show a broad range of variation [1], [2]. Infraorbital ethmoid cells are frequently found and are commonly known as “Haller cells” [3]. These cells may be an incidental finding or cause different disorders [12], [13], [16], [17], [18], [19], [22], [45], [49]. They are of special interest before sinus surgery, as they can lead to serious complications [14], [15], [49].

Haller cells have often been investigated in CT studies [15], [16], [17], [25], [45], [46], but CBCT studies are rare, as this is a new technique in sinunasal imaging [35]. We carried out a CBCT study to determine its ability to detect and measure variations in the region of the ethmoid complex. We found no correlation between the frequency or morphology of Haller cells, and age or gender. We introduced volumetry of the orbitoethmoid cell and present values for total and partial volume of this entity.

Second, we compared findings on OPG images to CBCT images and found no strong congruence between techniques, leading us to conclude that OPG is not suitable for diagnosing the presence of Haller cells. This result is contrary to former surveys [37], [38], [39]. As CBCT delivers detailed information on the ethmoid complex with lower costs compared to CT and the possibility of prompt information by implementation in an outpatient office [28], [29], we see further need for radiological investigation and clinical studies on Haller cells.

## Notes

### Competing interests

The authors declare that they have no competing interests.

### Authorship

Reinhard E. Friedrich and Meike Fraederich equally contributed to the article as joint first authors.
